# Targeting Oncogenic Activity and Signalling of Mutant Receptor Tyrosine Kinase FLT3

**DOI:** 10.3390/cancers17172931

**Published:** 2025-09-07

**Authors:** Boban Dobrevski, Hannah Willems, Carolin Lossius-Cott, Jörg P. Müller

**Affiliations:** 1 Institute of Molecular Cell Biology, Center for Molecular Biomedicine, University Hospital of Friedrich Schiller University, 07745 Jena, Germany; boban.dobrevski@med.uni-jena.de (B.D.); hannah.willems@med.uni-jena.de (H.W.); carolin.lossius@med.uni-jena.de (C.L.-C.); 2 Institute of Immunobiology and Human Genetics, Faculty of Medicine, Ss. Cyril and Methodius University in Skopje, 1000 Skopje, North Macedonia

**Keywords:** receptor tyrosine kinase (RTK), acute myeloid leukaemia (AML), FLT3 ITD, oncogenic mutations, protein tyrosine phosphatases (PTPs), alternative treatments

## Abstract

Fms-like tyrosine kinase 3 (FLT3) is a receptor tyrosine kinase (RTK) that is involved in cell survival, proliferation, and differentiation of haematopoietic progenitors of lymphoid and myeloid lineages. Oncogenic mutations in the FLT3 gene, resulting in constitutively active FLT3 variants, are frequently found in patients with acute myeloid leukaemia (AML). In particular, patients expressing FLT3 ITD (internal tandem duplications of the juxtamembrane domain of FLT3) correlate with poor patient survival. Targeting FLT3-mutated leukaemic stem cells is therefore a key to the efficient treatment of patients with relapsed/refractory AML. The efficacy of approved tyrosine kinase inhibitors is regularly compromised by various resistance pathways or secondary mutations. Based on the current molecular understanding of aberrant signal transduction pathways and cell transformation, novel alternative treatment approaches can be exploited for therapeutic purposes. In particular, new insights into the regulation of the activity of counteracting protein tyrosine phosphatases (PTPs), the aberrant biogenesis and activation of mutant FLT3 proteins, as well as common factors controlling cell transformation are attractive avenues. This review summarises the current knowledge about the regulation of the oncogenic activities of mutant FLT3 proteins and discusses possible options for alternative treatments.

## Simple Summary

Fms-like tyrosine kinase 3 (FLT3) is a receptor protein kinase that channels haematologic cell differentiation. Its gene is frequently mutated in acute myeloid leukaemia (AML) patients. One common mutation, FLT3 ITD, results in a constitutive active kinase, which promotes oncogenic cell proliferation and blocks cell differentiation. The occurrence of FLT3 ITD mutations is linked to worse outcomes. Current treatments target the FLT3 ITD mutation but often become ineffective due to the development of resistance or new mutations. We and others are exploring new treatment options by in-depth examination of how FLT3 ITD works and how its activity is controlled. This information is then used to abrogate its abnormal activity and consequently stop disease development.

## 1. Introduction

Fms-like tyrosine kinase 3 (FLT3), a member of the class III receptor tyrosine kinase family, is expressed in haematopoietic progenitor cells. As a receptor tyrosine kinase, FLT3 is part of the balanced process of protein tyrosine phosphorylation and plays a role in cell survival, proliferation, and differentiation. It is involved in the maintenance of pluripotent haematopoietic stem cells (HSCs) and contributes to the proliferation and differentiation of B-cell progenitors, myelomonocytic cells, and dendritic cells [1,2,3]. Overexpression of the wild type (WT) or oncogenic forms of FLT3 is closely associated with the development of acute myeloid leukaemia (AML). FLT3 mutations are among the most common genetic abnormalities in AML with approximately 23% of all AML patients exhibiting FLT3 mutations with internal tandem duplications (ITDs). These duplications of amino acid stretches in the juxtamembrane domain of the protein disrupt the auto-inhibition of its kinase activity, leading to ligand-independent dimerisation and signalling [4,5]. FLT3 ITD was identified as an unfavourable prognostic factor for achievement of complete remission, relapse-free survival, and overall survival of AML patients [6]. Mutations in the tyrosine kinase domain (TKD) account for about 7% of all AML cases [7]. TKD mutations are predominantly found in the activation segment of the kinase domain, most frequently observed at amino acids D835 and I836, with the substitution D835Y being the most common TKD mutation [8,9,10]. They result in the stabilisation of an active conformation of the activation segment. Consequently, mutant FLT3 proteins, especially FLT3 ITD, are potent oncogenic drivers in AML. By expanding the self-renewal of early progenitor cells, blocking HSC differentiation, and exploiting clonal dominance, they promote leukaemic transformation. Therefore, FLT3 proteins are the most important therapeutic target for mutant FLT3 AML [11]. An overview about here discussed treatment options targeting FLT3 ITD activity directly (Table 1) or FLT3 ITD-mediated oncogenic signalling (Table 2) is given.

## 2. Specific Targeting of FLT3 Activity with Tyrosine Kinase Inhibitors (TKIs)

In recent years, significant progress has been made in the development of tyrosine kinase inhibitors (TKIs) aiming at constitutively active FLT3 and its downstream signalling. Currently, three FDA-approved agents targeting mutant FLT3 are available for AML therapy [37,38,39]. First, midostaurin received approval for newly diagnosed FLT3-mutated AML in April 2017 [38] and is currently used for both classes of FLT3 mutations in newly diagnosed AML. Second, gilteritinib was FDA-approved for the treatment of relapsed or refractory AML with an FLT3 mutation in 2018. It is a second-generation TKI that inhibits several kinases, including FLT3, AXL, ALK, and c-kit [40]. Gilteritinib uniquely targets both the ITD and TKD mutations, including FLT3 ITD, FLT3 ITD-D835Y, and FLT3-D835Y [41]. Third, the FDA approved quizartinib in 2023, in combination with standard cytarabine and anthracycline induction and cytarabine consolidation, and as maintenance monotherapy following consolidation chemotherapy for the treatment of FLT3 ITD-positive AML patients. Quizartinib is a second-generation type 2 FLT3 inhibitor, which maintains anti-leukaemic activity in preclinical models of RAS-mediated midostaurin-resistant AML cells [42].

The availability of FLT3 inhibitors has continued to grow (recently reviewed by Rataj et al., 2025 [12]). Clinical trials continue to demonstrate the efficacy of this class of agents, with an expanding number available for both experimental and standard-of-care usage. Novel but not yet approved inhibitors such as crenolanib, originally developed as a platelet-derived growth factor receptor (PDGFR), overcome resistance mutations, including potent and selective inhibition of both ITD and D835 mutations [43].

Despite positive effects, TKIs cannot cure AML. Due to its particularly aggressive nature, AML still shows the lowest survival rate among the most common forms of leukaemia. Nearly 80% of those diagnosed with AML today will not survive beyond five years after diagnosis. Consequently, stem cell transplantation resulting in a 50–70% overall survival is still the preferred way to treat and cure AML, but not all patients are eligible for this treatment. Various resistance pathways such as the acquisition of secondary TKD mutations (e.g., D835, F691L), upregulation of alternative survival pathways (e.g., RAS/MAPK), and microenvironment-mediated protection abolish the mode of action of FLT3 TKI treatment [44], as reviewed by Müller and Schmidt-Arras 2020 [45]. An increasing number of combination strategies (e. g., gilteritinib and venetoclax) are currently designed to overcome this (for more details, see TKI on FLT3; [46]). Still, further insight into controlling the activity and signalling of mutant FLT3 is the prerequisite to further improve treatment options for AML patients. In this article, we review alternative approaches to affect oncogenic FLT3 signalling entities in particular.

TKI-mediated inhibition of FLT3 ITD signalling in leukaemic blast cells can be bypassed by the induction of expression and cytokine-mediated activation of other RTKs, such as the receptor tyrosine kinase Axl, G-CSFR, or by activation of the interleukin (IL)-3 receptor complex. Thus, convergent signals from the haematopoietic microenvironment drive FLT3 ITD cell resistance. It has been demonstrated that FLT3-ITD-expressing leukaemic cells develop TKI-resistance via enhanced expression of Axl [47,48]. Activation of Axl most likely occurs via autocrine secretion of its ligand Gas6. Therefore, dual targeting of FLT3 and Axl seems to be a promising strategy to overcome FLT3 inhibitor resistance in FLT3 ITD-positive AML. Combinations of AXL-targeted agents such as bemcentinib [49] or TP-0903 [50] with FLT3 TKI exerted synergistic cytotoxic effects and induced apoptosis in FLT3 ITD cells and FLT3 inhibitor-resistant blast cells. Preclinical investigations of cabozantinib, targeting VEGFR, MET, AXL, KIT, or RET, in combination FLT3 inhibition helped to overcome FLT3 inhibitor resistance by targeting, in particular, alternative signalling pathways involved in angiogenesis, stromal interactions, etc. [13]. Further, when microenvironmental signalling is prominent, dasatinib, an SRC-family and multi-kinase inhibitor (targeting SRC, ABL, KIT, and PDGFR), overcomes bone marrow stroma–based resistance to selective FLT3 inhibitors by blocking extrinsic STAT5/STAT3 activation [51]. By using ibrutinib, an inhibitor of the RTK BTK, it has been demonstrated that concomitant targeting of FLT3 and BTK overcomes FLT3 inhibitor resistance in AML through the inhibition of autophagy [52]. A phase I trial with mivavotinib (TAK-659), a dual SYK/ FLT3 inhibitor, in patients with relapsed/refractory AML revealed disrupted SYK-driven FLT3-independent support as well as activity in FLT3-mutant subsets of patients [53]. FLT3 TKI resistance frequently rides the RAS/MAPK network. While MEK/ERK or SHP2 agents are not tyrosine kinase inhibitors, they are often combined with FLT3 TKIs to suppress these bypass routes and restore sensitivity [54]. Allosteric SHP2 inhibition increases apoptotic dependency on BCL2 and synergises with venetoclax in *FLT3-* and *KIT-*mutant AML [54].

Despite the wide layer of approaches to counteract the oncogenic signalling of FLT3 ITD directly or indirectly, none of the approaches can completely eradicate oncogenic cell clones. In order to improve the outcome of FLT3 ITD-positive AML therapies, combination approaches targeting different aberrant activities of the mutant cell clones are under development. In particular, the current standard drug combination with cytrabine and daunorubicin or cytrabine and anthracycline (CPX-351) in a 7 + 3 modus [55,56] is frequently combined with midostaurin or other TKIs. Particularly for older or unfit patients, triplet therapies combining hypomethylating agents (e.g., azacitidine) or the BCL-2 inhibitor venetoclax with FLT3 inhibitors (quizartinib or gilteritinib) show encouraging early results [57]. Combinatory therapies require careful scheduling to manage profound myelosuppression. Several phase III and larger randomised studies are ongoing and will determine practice in the next couple of years.

Taken together, the above data reveal that the further development of combinatory kinase inhibition strategies has a high potential to overcome resistance against FTL3-specific TKIs (Table 3). While combinatory therapies with FLT3 inhibitors and standard chemotherapy are routinely used to treat FLT3 ITD-positive AML patients in particular, combinatory multi-target kinase inhibition awaits approval.

## 3. Mediating FLT3 Activity by Controlling Its Maturation and Degradation

### 3.1. Targeting Glycosylation and Plasma Membrane Localisation

**Figure 1:**
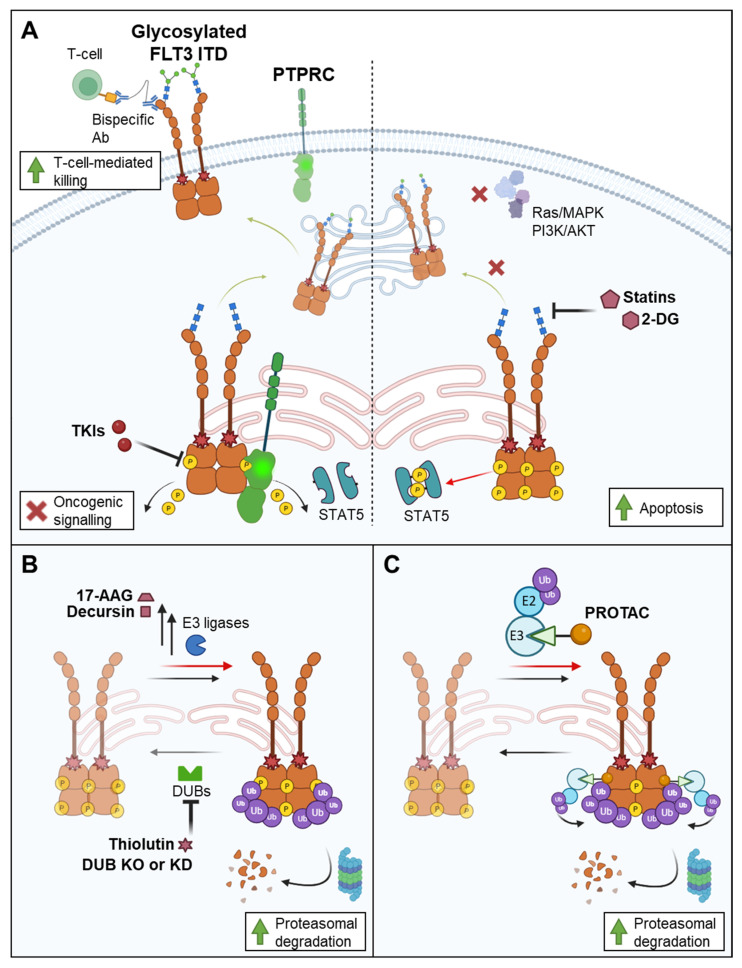
Targeting FLT3 ITD by addressing glycosylation and inducing degradation. (**A**) Targeting FLT3 ITD biogenesis and glycosylation. Left: Following protein synthesis, FLT3 ITD is folded in a chaperone-assisted manner and glycosylated in the endoplasmic reticulum (ER, shown as the compartment with the red membrane) forming an immature mannose-rich protein with a molecular weight of about 130 kDa (blue quadrants on the FLT3 molecule). FLT3 ITD is predominantly present in this 130 kDa high-mannose form in the ER and Golgi. In the case of FLT3 WT, this immature form is further processed in the Golgi apparatus to its mature 150 kDa complex-glycosylated form (extension with blue quadrants and green circles). Via vesicular transport, FLT3 then moves to the plasma membrane to form a functional ligand-accessible receptor. A similar biogenesis route can be achieved for oncogenic FLT3 ITD by abrogation of kinase activity by tyrosine kinase inhibitors (TKIs) or increased activity of counteracting phosphatases (exemplified by PTPRC). The dephosphorylated receptor undergoes complex glycosylation and is translocated to the plasma membrane similar to the WT form. The membrane-localised FLT3 ITD is accessible to FLT3-CD3 bispecific antibodies or similar entities, facilitating T-cell-mediated cytotoxicity. Right: Treatment with 2-deoxy-D-glucose (2-DG) or statins impairs receptor glycosylation and partly increases STAT5 activation. Inhibition of glycosylation retracts receptor plasma membrane localisation and inhibits RAS/MAPK and PI3K/AKT signalling resulting in induction of apoptosis. (**B**) Induction of FLT3 ITD proteasomal degradation. FLT3 undergoes reversible ubiquitination labelling it for proteasomal degradation. Ubiquitination and deubiquitination are mediated by members of the E3 ligase family and deubiquitinating enzymes (DUBs), respectively. Enhanced receptor ubiquitination and degradation is achieved by increased expression or activity of E3 ligases, e.g., by decursin or 17-AAG (17-allylaminodemethoxygeldanamycin) treatment. Inhibition of DUBs using knockdown (KD) or knockout (KO) approaches or by thiolutin also promotes receptor ubiquitination and degradation. (**C**) Degradation of FLT3 ITD by protein-targeting chimeras (PROTACs). Bifunctional small molecules (PROTACs) recruit E3 ligases to the FLT3 receptor resulting in receptor ubiquitination and proteasomal degradation.

During biogenesis, RTKs undergo complex glycosylation before reaching the plasma membrane (Figure 1A): Following protein synthesis, FLT3 is folded in a chaperone-assisted manner and then glycosylated in the endoplasmic reticulum (ER) to form an immature mannose-rich glycosylated protein with a molecular weight of about 130 kDa. This immature FLT3 is further processed in the Golgi apparatus to its mature 150 kDa complex-glycosylated form [61,62,63]. Via vesicular transport, FLT3 moves to the plasma membrane to form a functional ligand-accessible receptor (Figure 1A). In contrast, oncogenic FLT3 ITD is predominantly present in a 130 kDa high-mannose form in the ER and Golgi. As a result, the trafficking of FLT3 ITD to the plasma membrane is impaired and the oncogenic receptor is retained in these intracellular compounds [61,62,64]. The molecular reason for the retention of constitutively active mutant FLT3 proteins is not known. Since pharmacologic or genetic abrogation of kinase activity results in dominant plasma membrane localisation, phosphorylation has been discussed as a retention factor [64]. The interaction of the serine/threonine kinase Pim-1 with the immature 130 kDa FLT3 ITD contributes to its stabilisation, which at least partially prevents complex glycosylation and maturation of the receptor and therefore results in retention of FLT3 ITD [65].

The subcellular localisation affects the downstream signalling of FLT3 (Figure 1A). Plasma membrane-localised FLT3 is activated by dimerisation and trans-autophosphorylation upon binding of the FLT3 ligand. This leads to the activation of the signalling cascades RAS-RAF-ERK1/2 and PI3K/AKT. In contrast, intracellularly retained FLT3 ITD phosphorylates and activates STAT5 ligand-independent signalling but shows reduced ERK1/2 and AKT activation [66,67]. The transport of FLT3 from the ER to the plasma membrane has not been addressed in detail. It might follow the common secretory route of RTKs via COPII-coated vesicles at the ER, the trans-Golgi network, and vesicular transport to the plasma membrane, where it is available for ligand binding. An siRNA-based RNAi screen utilising a STAT5-driven reporter assay in FLT3 ITD-expressing cells was enriched for genes encoding proteins involved in protein secretion and intracellular protein transport, indicating that modulation of protein transport processes could potentially be used to reduce aberrant STAT5 signalling in FLT3 ITD-positive cells [67]. The downregulation of the key retrograde transport receptor KDELR1, involved in the Golgi to ER protein traffic, results in increased FLT3 ITD surface localisation and reduced cell proliferation and colony formation. In addition, the capacity to generate leukaemia-like disease after transplantation was reduced [68]. Marcotegui et al. described SET, a scaffolding protein, as relevant for FLT3 trafficking and plasma membrane localisation. SET co-localises with FLT3 WT but not with FLT3 ITD and facilitates FLT3 WT trafficking to the plasma membrane. The interaction of FLT3 and SET occurs prior to glycosylation, and tyrosine kinase inhibition by midostaurin increases SET binding and FLT3 surface localisation. The impaired binding of SET to FLT3 ITD contributes to its intercellular retention [18].

By directly targeting the glycosylation status of FLT3, its localisation can be influenced, which subsequently would result in changes in its downstream signalling. It has been demonstrated that the abrogation of RTK glycosylation would result in impaired oncogenic activity of mutant FLT3 proteins (Figure 1A). Statins block 3-hydroxy-3-methylglutaryl coenzyme A reductase (HMGCoA reductase), which is important for the generation of dolichol via the mevalonate pathway. Dolichol is linked to the transfer of oligosaccharides to polypeptides that undergo N-linked glycosylation [69,70]. Treatment of FLT3 ITD cells with fluvastatin, a clinically applied statin that interferes with the mevalonate synthesis as described above, leads to further impairment of glycosylation and consequently results in a loss of surface localisation. The forced FLT3 ITD retention was associated with increased STAT5 activation but inhibition of both MAPK and AKT phosphorylation, consequently resulting in induction of apoptosis [15]. In addition, reduced engraftment of FLT3 ITD BaF3 cells revealed diminished oncogenicity of FLT3 ITD after fluvastatin treatment [15]. Furthermore, the combinatory treatment of fluvastatin with TKI was shown to have synergistic effects in inhibiting growth and promoting apoptosis of FLT3 ITD-positive leukaemic cells [17].

2-Deoxy-D-glucose (2-DG) is a potent inhibitor of the glycolytic pathway, shown in several solid tumours [16,71,72]. Additionally, 2-DG inhibits N-linked glycosylation independently of its effects on glycolysis [73,74]. In FLT3 ITD-expressing cell lines and AML samples, 2-DG treatment leads to tumour cell growth inhibition and induces apoptosis. FLT3 surface expression and ERK1/2 as well as STAT5 phosphorylation are reduced by 2-DG due to inhibition of N-linked glycosylation. Tsitsipatis et al. showed that inhibition of N-linked glycosylation by tunicamycin abolishes FLT3 ITD glycoprotein maturation and activation of STAT5. Low doses of tunicamycin had anti-proliferative and pro-apoptotic effects on FLT3 ITD-expressing human and murine cell lines through the activation of protein kinase RNA-like endoplasmic reticulum kinase (PERK) and activation of the gene encoding CCAAT-enhancer-binding protein homologous protein (*CHOP*). In addition, the synergistic effects of tunicamycin and TKI treatment further inhibited the cell viability of FLT3 ITD-expressing cell lines and primary AML cells [17].

In contrast, inhibition of FLT3 activity by inactivating point mutations, small molecules (TKIs), or co-expression of PTPs (see below) forces complex glycosylation and plasma membrane localisation (Figure 1A, [19,61,64,75]. Surface-localised mutant FLT3 in TKI-treated cells can be targeted by bispecific FLT3-CD3 antibodies for T-cell-mediated cytotoxicity, shown to be effective in FLT3 ITD-positive AML cell lines, patient-derived xenograft cells, and primary patient samples [19]. Alternatively, surface localisation of FLT3 ITD was also significantly enhanced after treatment with the histone deacetylase inhibitor valproic acid [20].

Taken together, the above data demonstrate that altered glycosylation impaired FLT3 ITD-mediated cell transformation in both ways. While the glycosylation inhibitor tunicamycin further attenuated plasma membrane localisation, it caused pronounced ER stress and apoptosis. On the other hand, improving cell surface localisation of FLT3 ITD by complex glycosylation causes the receptor to be accessible for cell surface-based treatment options and reduces oncogenic STAT5 signalling.

### 3.2. Targeting Degradation Through the Ubiquitin Pathway

FLT3 activity can also be regulated by its lysosomal and proteasomal degradation, which is mediated by ubiquitination [24,63]. Ubiquitination is a reversible biological process regulated by ubiquitin ligases and deubiquitinating enzymes (DUBs) [63,76]. FLT3 ligand binding and activation triggers FLT3 ubiquitination, which is facilitated by multiple ubiquitin ligases at multiple ubiquitination sites. Polyubiquitinated FLT3 is internalised and subsequently degraded [63,77,78,79]. Promoting ubiquitination and degradation of the receptor reduces FLT3 ITD signalling and oncogenic activity (Figure 1B).

Members of the suppressor of cytokine signalling (SOCS) family of E3 ubiquitin ligases are known to regulate the signalling of cytokine receptors and RTKs. Two members of this family, SOCS6 and SOCS2, have been implicated in FLT3 ubiquitination and thus as regulators of FLT3 signalling [78,80]. Upon FLT3 ligand stimulation, SOCS6 binds to FLT3 and enhances FLT3 ubiquitination, internalisation, and degradation while simultaneously reducing ERK1/2 signalling in several cell lines. The absence of SOCS6 promotes proliferation in FLT3 ITD-expressing cell lines, demonstrating that SOCS6 is able to regulate FLT3 WT and FLT3 ITD signalling [78]. Similarly, SOCS2 increases FLT3 ubiquitination and degradation and negatively regulates FLT3 signalling via ERK1/2 and STAT5. In addition, SOCS2 expression leads to a decrease in FLT3 ITD-mediated cell proliferation [80]. CBL, another E3 ubiquitin ligase, facilitates the polyubiquitination of FLT3 ITD, specifically at lysine residue 48 (K-48), leading to proteasomal degradation of the receptor. Thus, CBL negatively regulates FLT3 activity [24,81]. Consistent with this, CBL exon 8/9 mutants are able to induce FLT3 autophosphorylation, STAT5 and AKT activation, and cell proliferation. Overexpression of loss-of-function CBL mutants increased FLT3 downstream activity [82]. Polyubiquitination of FLT3 ITD at lysine 63 (K-63) can occur via the E3 ubiquitin ligase NEDD4 [24].

One way to enhance FLT3 ubiquitination is to inhibit deubiquitination and DUBs (Figure 1B). Deubiquitination at lysine 48 is mediated by a DUB called USP10 [22]. Inhibition of USP10 leads to increased FLT3 degradation with efficacy in FLT3 ITD-AML models such as cell lines, primary AML samples, and mouse models [22]. Another DUB, USP9X, is known to associate with FLT3 ITD and inhibit lysine 63-linked ubiquitination [83]. Inhibition of USP9X enhances lysine 63-linked ubiquitination of FLT3 ITD and has pro-apoptotic effects in cells expressing FLT3 mutants, including FLT3 ITD-positive AML patient samples. Downregulation of USP9X reduces the expression levels of FLT3 ITD and its downstream signalling [83]. Another lysine 63-linked DUB, BRCA1/BRCA2-containing complex subunit 36 (BRCC36), binds exclusively to FLT3 ITD and promotes protein stability and activation by hydrolysing lysine 63-linked polyubiquitin chains. Knockdown of BRCC36 reduces FLT3 ITD-mediated STAT5 phosphorylation and cell proliferation in FLT3 ITD-expressing cells [84]. In addition, cell proliferation is suppressed in FLT3 ITD-expressing cells by inhibition of DUB BRCC36 with the sulfur-containing antibiotic thiolutin. Thiolutin treatment induces apoptosis and shows synergistic effects with TKIs to reduce the cell viability of FLT3 ITD-expressing cell lines (Figure 1B) [84]. A recent study revealed that decursin, a pyranocoumarin natural product extracted from *Angelica gigas* Nakai root, impaired the cell viability of FLT3 ITD-positive AML cells and cell lines [23]. In addition, decursin is able to alleviate leukaemia burden in mouse models. These effects are based on increased expression of the E2-conjugating enzyme UBCH8/UBE2L6 due to decursin treatment. Increased expression of UBCH8/ UBE2L6 leads to FLT3 ITD ubiquitination, proteasome-mediated degradation, and cell apoptosis [23,77]. Zhang et al. further showed the synergistic effects of decursin and venetoclax to induce apoptosis in FLT3 ITD-expressing cell lines [23].

Furthermore, FLT3 ITD ubiquitination and degradation have been shown to be induced by treating cells with the heat shock protein 90 (HSP90) inhibitor 17-allylamino demethoxy geldanamycin (17-AAG, Figure 1B). HSP90 is a molecular chaperone for correct protein folding of FLT3 [85]. Treatment with 17-AAG induces the CBL-mediated polyubiquitination of FLT3 ITD, leading to its proteasomal degradation in leukaemic cells [24].

These results show that the ubiquitination process of FLT3 can be influenced directly but also indirectly, e.g., via HSP90 inhibition or histone deacetylation (see below). Increasing FLT3 ubiquitination and proteasomal degradation is an efficient but not very specific way to target its activity.

### 3.3. Targeting Degradation by PROTACs

PROTACs (proteolysis targeting chimeras) are bifunctional molecules that recruit E3 ubiquitin ligase to target proteins, leading to their ubiquitination and degradation by the proteasome (Figure 1C). These small molecules work by hijacking the cell’s natural protein degradation system to selectively eliminate disease-related proteins. PROTACs are heterobifunctional molecules consisting of a ligand (e.g., a small-molecule inhibitor of the target protein) that is covalently linked to an E3 ubiquitin ligase ligand. The mechanism of action involves the formation of a ternary complex between the protein of interest (e.g., FLT3 ITD), PROTAC, and an E3 ligase that causes FLT3 ITD to be ubiquitinated and subsequently degraded [86,87].

The application of PROTACs targeting FLT3 has advantages over the use of traditional FLT3 inhibitors because due to the degradation of the receptor, it not only leads to abrogation of oncogenic signalling but also to the blockade of non-kinase functions. Kinase-inhibited mutant FLT3 proteins can promote leukaemogenesis by acting as scaffolds in signalling complexes such as interactions with adaptor proteins and stabilising oncogenic complexes. In addition, PROTACs can target multiple FLT3 proteins, and thus, the formation of secondary resistance mutations (such as D835Y or F691L) is similarly removed. Due to their specificity, they avoid off-target effects common to ATP-competitive inhibitors that hit other kinases. This consequently allows for lower dosing and potentially fewer off-target toxicities (Figure 1C). A quizartinib-based PROTAC has been shown to increase FLT3 ITD degradation with subsequent enhanced anti-proliferative cellular activity and selectivity in mouse models [25]. Similarly, the multi kinase inhibitor dovitinib was modified to recruit the E3 ubiquitin ligase cereblon (CRBN) to FLT3 ITD. Consequently, it exhibited anti-proliferative effects against FLT3 ITD AML cells in vitro and in vivo by inducing the degradation of FLT3 ITD and c-KIT, thereby abolishing oncogenic downstream signalling [88]. Pomalidomide, a bispecific PROTAC targeting both FLT3 and CDK9, has been shown to attack FLT3 ITD-bearing cells by abrogating FLT3 ITD downstream signalling and inducing cell cycle arrest [88,89]. Several other PROTACs targeting FLT3 alone have also been shown to degrade the kinase, reduce oncogenic FLT3 ITD signalling, and decrease cell proliferation in vitro, as well as exhibiting anti-tumour activity in vivo [21,31,87,90,91]. The combination of PROTACs with other treatments that are already approved for AML is also under investigation. Combining a gilteritinib analogue PROTAC targeting FLT3 ITD with venetoclax demonstrates increased synergistic anti-AML effects in vivo, with lower normal tissue toxicity compared to gilteritinib and venetoclax together. This highlights a way to overcome toxicity problems in clinical trials [60].

Overall, targeting the stability of FLT3 by modulating its ubiquitination pharmacologically represents a promising therapeutic strategy for FLT3 ITD-positive AML patients. Promoting the degradation of the oncogenic FLT3 receptor via enhanced ubiquitination pathways may effectively reduce leukaemic cell proliferation, overcome drug resistance issues, and improve treatment outcomes.

## 4. Controlling FLT3 Trans-Autophosphorylation by Complex Formation

A prerequisite for RTK phosphorylation and subsequent activation is its trans-autophosphorylation. In general, the resting receptor is located in the cell membrane as an auto-inhibited monomer. Binding of its related ligand results in a conformational change of the receptor ectodomain mediating dimeric receptor assembly. Consequently, the dimerisation allows the adjacent intracellular tyrosine kinase domains to become trans-activated by auto-phosphorylation. The phosphorylated tyrosine site forms the platform for interaction of the SH2 domains of adaptor proteins activating downstream signalling [80,92]. In particular, the growth factor receptor binding protein-2 (GRB2), which couples RTKs to the MAPK signalling pathway, interacts with FLT3 tyrosines 768, 955, and 969. The association of the scaffolding protein GRB2 Associated Binding Protein 2 (GAB2), which in turn directly interacts with PI3K, facilitates activation of the AKT signalling pathway [93]. 

Regardless of the sequence variety of the JM domain, FLT3 ITD receptors also form homodimers in the absence of FL and, if co-transfected with FLT3 WT, FLT3 WT/FLT3 ITD heterodimers [4,14]. Thus, elongation mutations of the JM domain promote FLT3 ITD receptor dimerisation and its subsequent phosphorylation and auto-activation in the absence of the ligand [4].

Gangliosides, which are glycosphingolipids containing one or more sialic acid residues, are natural integral components of the outer leaflet of the plasma membrane of mammalian cells. Their amphipathic structure allows them to participate in various cellular processes, especially in membrane microdomains such as lipid rafts [94]. Lipid rafts are enriched with cholesterol and sphingolipids. They play critical roles in cellular processes by organising and concentrating specific proteins. Thus, lipid rafts promote protein–protein interaction and consequently influence receptor and protein dimerisation and stabilise signalling complexes (recently reviewed in [95]). Several studies addressed the role of gangliosides in regulating RTK activity. It has been demonstrated that the GM1 ganglioside interacts with the Trk neurotrophin receptor facilitating its ligand-induced dimerisation, activation, and subsequently downstream signalling ([96,97,98], reviewed in [26]). By modulation of endogenously expressed gangliosides, the dimerisation and activation of EGFR are altered [99]. In addition, it is known that cancer cells alter ganglioside composition to favour receptor dimerisation and signalling as demonstrated for Her2 (ErbB2) and EGFR [100,101]. In human glioma, application of gangliosides resulted in inhibition of PDGFR dimerisation and corollary phosphorylation [102]. Thus, external application of gangliosides would be an attractive alternative way to interfere with RTK complex formation and its trans-autophosphorylation. So far, the effects of gangliosides on FLT3 dimerisation have not been studied yet.

## 5. Regulation of RTK Activity by Reactive Oxygen Species (ROS)

Activation of RTKs leads to the production of reactive oxygen species (ROS) such as hydrogen peroxide (H_2_O_2_) by membrane-bound NADPH oxidases. ROS can oxidise specific cysteines in RTKs. Mild oxidation results in the formation of disulfide bonds or sulfenic acid modifications, which can stabilise active conformations or modulate the receptor’s activity. Thus, ROS are important mediators in the physiological regulation of cell functions and in the context of different pathologies [103,104]. Elevated ROS levels can directly alter kinase function via cysteine oxidation as it was demonstrated for PDGFR [105] and EGFR [106]. It has been shown that the EGFR active-site cysteine (C797) is the target of oxidation. Its sulfenylation mediates a new electrostatic interaction with the catalytic loop. Chronic oxidative stress yields in an oxidized EGFR population, which was refractory to its TKI afatinib [107].

Comprehensive analysis of cysteine-to-serine mutant FLT3 ITD proteins revealed critical roles of several cysteine residues for the kinase’s activity, signal transduction, and cell transformation, further supporting cysteine modification as a potential mechanism of activity regulation [14]. Importantly, these effects were not related to altered FLT3 ITD dimerisation but likely caused by changed intramolecular interactions. Consistently, treatment of cells expressing FLT3 ITD with ROS-quenching agents attenuated signal transduction [14]. These findings identify the functional relevance of all cytoplasmic FLT3 ITD cysteines and indicate the potential for redox regulation of this clinically important oncoprotein. 

## 6. Controlling FLT3 Activity by Regulating the Activity of Protein Tyrosine Phosphatases (PTPs)

PTPs work in coordination with protein tyrosine kinases to regulate cellular signalling pathways, often counteracting RTK activity by dephosphorylating tyrosine residues [108]. Non-receptor PTPs are localised in the cytoplasm or nucleus, while receptor-type PTPs (RPTPs) are membrane-bound and possess extracellular domains similar to immunoglobulins and fibronectin type III. These RPTPs regulate processes such as cell adhesion, migration, and differentiation and play important roles in development and immune responses [109]. 

**Figure 2:**
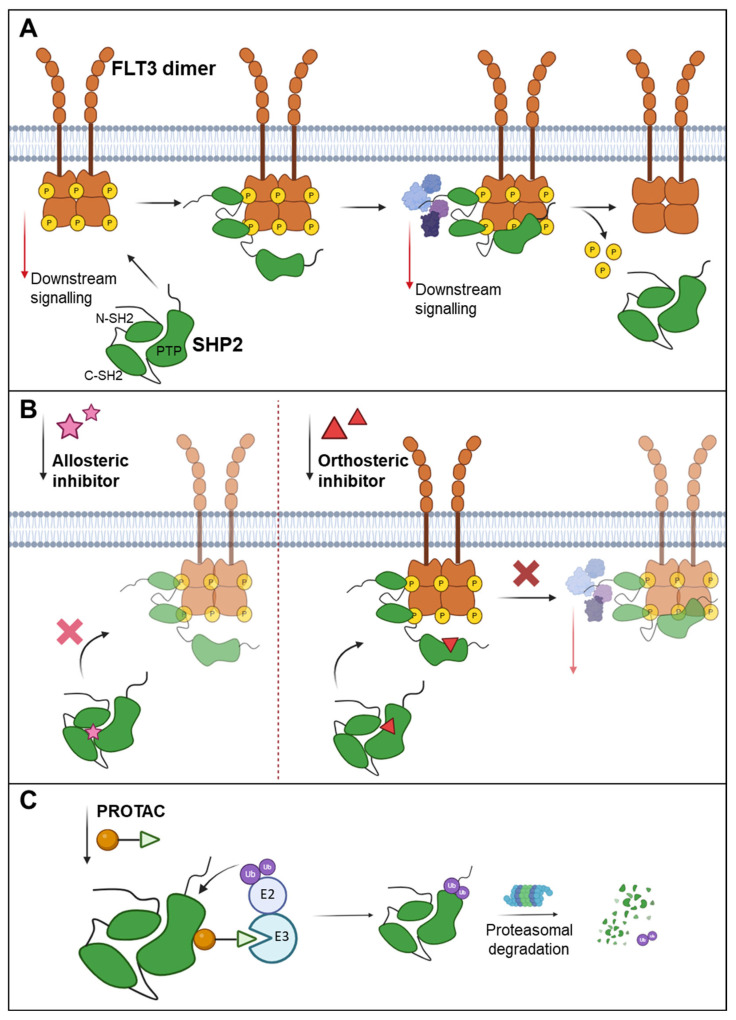
Inhibition of pro-oncogenic FLT3 interacting PTPs, exemplified by SHP2. (**A**) SHP2 is recruited to the phosphorylated FLT3 receptor by SH2 domains recognising the phosphorylated tyrosine site at FLT3. While the PTP domain dephosphorylates the FLT3 receptor, the N-SH2 domain tail serves as a docking platform for signalling proteins involved in the RAS/MAPK, PI3K/AKT, and STAT5 pathways, thereby facilitating downstream signal transduction. (**B**) Inhibition of SHP2. Stabilisation of inactive SHP2 by allosteric inhibitors (left). Orthosteric PTP inhibitors target the catalytic phosphatase site (right). (**C**) Targeting SHP2 for degradation using protein-targeting chimeras (PROTACs). Binding of PROTACs to the target phosphatase, for example, SHP2, facilitates its degradation by harnessing the ubiquitin-proteasome system.

PTPs are key regulators, both positive and negative, of FLT3 kinase activity, which influences cell proliferation, differentiation, and survival. For example, SHP2 (encoded by *PTPN11*) enhances FLT3 ITD-driven STAT5 signalling, contributing to the hyperproliferation of haematopoietic progenitors and the development of malignancies (Figure 2A) [32]. Germline mutations in PTPN11 were first observed in Noonan syndrome [110,111], while somatic mutations have been identified in myeloid malignancies such as juvenile myelomonocytic leukaemia (JMML), myelodysplastic syndromes (MDSs), and acute myeloid leukaemia (AML) [110,112,113]. The shRNA-mediated downregulation of SHP2 expression revealed a stimulating role in FLT3 WT-expressing cells [114]. In addition, an attenuating role of PTPN11 depletion on FLT3 ITD-mediated signalling has been demonstrated by Nabinger and co-workers. Thus, SHP2 positively contributes to FLT3 ITD-induced haematopoietic progenitor hyperproliferation and malignant disease in vivo [32]. 

**Figure 3:**
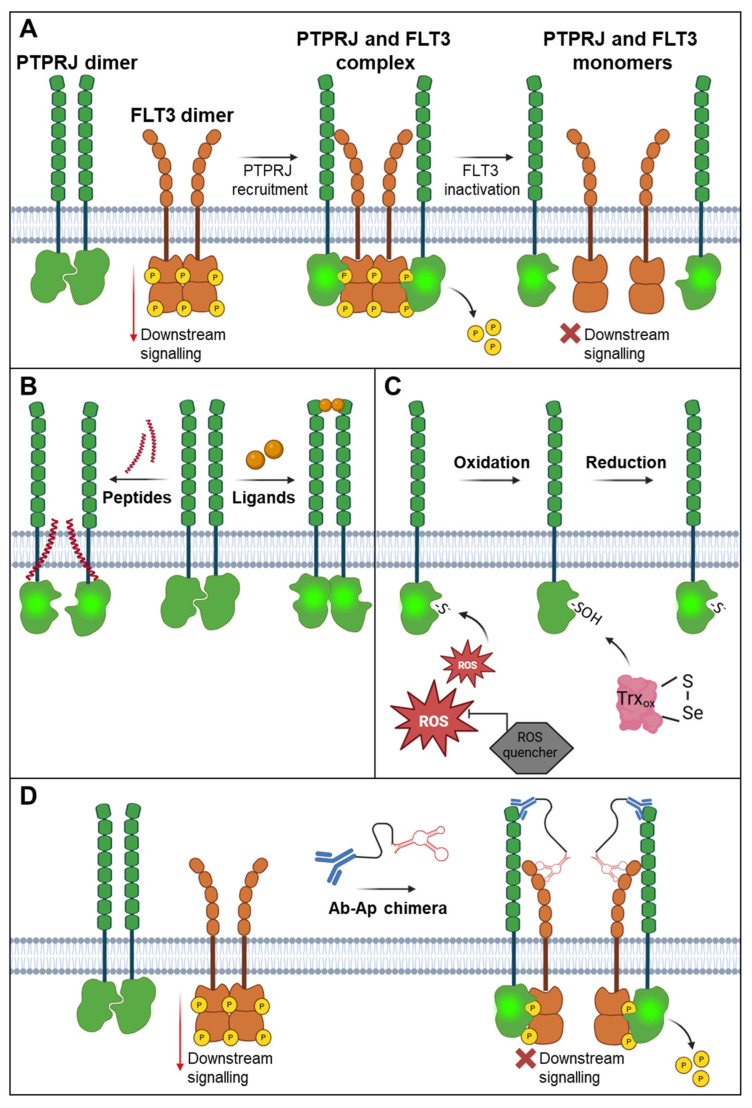
Activation of FLT3 antagonising protein tyrosine phosphatases (PTPs), exemplified by PTPRJ. (**A**) FLT3-PTPRJ complex formation. PTPRJ activity is impaired due to homodimerisation. Active monomeric PTPRJ (in glowing green) is recruited to the active dimeric FLT3 and dephosphorylates it. This results in receptor inactivation and reduced downstream oncogenic signalling. (**B**) Activation of PTPRJ by disruption of homodimerisation. Enhanced PTPRJ activity can be achieved by interfering complex formation using transmembrane peptides or ligand binding, which disrupts the wedge insertion mechanism that mediates its inactivation. (**C**) Prevention of PTPRJ oxidation. FLT3-mediated production of reactive oxygen species (ROS) leads to the oxidation of the catalytic cysteine of PTPRJ (−SOH). Endogenous thioredoxin (Trx_ox_) or ROS quenching molecules restore PTP activity by reducing the catalytic cysteine (−S^−^). (**D**) Antibody–aptamer chimeras inducing RPTP receptor contact. Bispecific antibody–aptamer chimeras recruit PTPRJ to membrane-localised receptor tyrosine kinase. Improved complex formation enhances receptor dephosphorylation. Schematic representation of a possible PTPRJ–FLT3–aptamer interaction.

Conversely, other PTPs negatively regulate FLT3 activity (Figure 3A). Overexpression of SHP1, PTP1B, and PTP-PEST dephosphorylates FLT3 ITD and promotes its maturation and surface localisation [61]. A systematic RNAi screen identified PTPRJ (DEP-1) and PTPRC (CD45) as negative regulators of FLT3 signalling [115]. Reduction or depletion of PTPRJ leads to increased FLT3 activation and enhanced FL-mediated signalling, proliferation, and clonal growth [115,116]. PTPRJ overexpression reduces FLT3 phosphorylation and downstream signalling. In addition, direct PTPRJ and FLT3 interaction and selective dephosphorylation of tyrosine residues controls FLT3 activity [115]. The antagonising role of PTPRC and PTPRJ on FLT3 ITD signalling in vivo has been shown in FLT3 ITD-expressing mice lacking PTPRJ or PTPRC. Here, FLT3 ITD-mediated haematologic aberrancies and myeloproliferative disorders were enhanced by KO of the PTP [117,118]. In addition, the involvement of FLT3 ITD and PTPRC in bone remodelling has also been demonstrated [119]. Since PTPs affect FLT3 oncogenic signalling, modulation of PTP activity, such as inhibition of proto-oncogenic PTPs or enhancing the activity of tumour-suppressive PTPs, can directly influence the phosphorylation and activity of FLT3 proteins. Thus, selective modulation of PTP activity is an attractive way to control the activity of FLT3 proteins.

### 6.1. Targeting PTPs by PROTACs

In contrast to RTK-inhibitory PTPs such as PTPRJ, several PTPs are known to stimulate oncogenic RTK signalling. To impair oncogenic effects, these PTPs can either be inhibited or degraded to reduce their activity (Figure 2C). Currently, there are not many studies focusing on PTP degradation by ubiquitination. However, the development of PROTACs is a promising approach to target pro-oncogenic PTPs, similar to the RTK targeting described above. The targeted degradation of PTPs offers several advantages over traditional inhibitors that in general block enzyme activity [120]. In general, PROTACs show increased potency due to the elimination of both catalytic and non-catalytic functions, especially for previously “undruggable” targets. They have the possibility to be highly selective and are hardly susceptible to resistance development by mutations [120].

The PROTAC DU-14 acts as a dual degrader, targeting both PTP1B and TC-PTP (PTPN2), PTPs that are negative regulators of T-cell activation and tumour antigen presentation. This PROTAC molecule consists of a PTP1B/TC-PTP active site-directed inhibitor DI-03 linked to an E3 ligase ligand and effectively induces the degradation of both PTPs with low nanomolar potency. This degradation enhances anti-tumour immunity by boosting IFN-γ-mediated JAK-STAT signalling, antigen presentation in tumour cells, and activation of CD8+ T-cells. DU-14 also inhibits tumour growth in immunocompetent mice with increased CD8+ T-cell infiltration [121]. TP1L is a PROTAC that selectively targets TC-PTP. Here, F_2_PMP-based TC-PTP inhibitor 3 was used to target this PTP. With over 110-fold selectivity for TC-PTP versus PTP1B, TP1L enhances IFN-γ signalling, increases the phosphorylation of JAK1 and STAT1, and boosts MHC-I expression without affecting PTP1B substrates such as JAK2. In T-cells, TP1L activates TCR signalling through elevated LCK phosphorylation, promoting T-cell proliferation and activation. It also activates CAR-T cells, enhancing their efficiency in killing tumour cells through both early and late T-cell activation, showing its potential in immune-based therapy development [122]. The first PROTAC targeting SHP2 was presented by Wang et al. [90]. It was based on the SHP2 inhibitor SHP099 (further discussed in Section 6.3). Considering the supportive role of SHP2 in FLT3 ITD oncogenic signalling, this PROTAC is of special interest in the context of targeting FLT3 activity [32]. This new SHP2-D26 PROTAC molecule was shown to be 30 times more potent in the inhibition of ERK phosphorylation and cell growth than SHP099, clearly showing the potential for the usage of PROTACs in drug development [90].

### 6.2. Orthosteric Inhibition of PTPs

To impair oncogenic kinase activity, RTK-stimulatory PTPs can be orthosterically inhibited by directly targeting the enzyme’s active site. This approach has been extensively explored for various PTPs, including PTP1B, SHP2, and TC-PTP [33,123,124] (Figure 2B). Direct targeting of their active site has historically been challenging due to the highly conserved catalytic domain centred around a cysteine residue, which makes selectivity hard to achieve. Thus, many inhibitors hit multiple PTPs.

One example for the specific orthosteric inhibition of a PTP is phenylhydrazonopyrazolone sulfonate (PHPS1), a cell-permeable and non-cytotoxic inhibitor, specific for SHP2. PHPS1 inhibits SHP2-dependent ERK1/2 signalling and thus cell proliferation and colony formation of human tumour cell lines (Figure 2B). Here, no off-target effects against SHP2-independent signalling could be observed [33].

However, in general, the rigid and positively charged nature of the active site further complicates the design of effective inhibitors with favourable pharmacokinetic properties [125]. Recent studies have highlighted the limitations of orthosteric inhibitors and emphasised the need for alternative strategies to overcome these challenges [126,127]. Consequently, efforts have increasingly shifted towards exploring allosteric inhibition mechanisms, which offer the potential for greater specificity and reduced adverse effects.

### 6.3. Allosteric Inhibition of PTPs

Mechanistically, allosteric inhibition stabilises inactive conformations by disrupting domain–domain interactions and preventing the WPD (Tryptophan-Proline-Aspartate) loop closure, which is essential for the phosphatase activity [128,129,130]. Since allosteric sites are less conserved compared to catalytic sites, allosteric inhibition results in improved selectivity, which also reduces the toxicity of the inhibitors by reduced interference with similar PTP family members. In addition, allosteric inhibitors are smaller, allowing higher specificity, better pharmacokinetics, and easier fine-tuning.

Selective allosteric PTP inhibition has been demonstrated for SHP2 as well as PTP1B. In SHP2, the catalytic PTP domain is followed by two SH2 domains. In the inactive (auto-inhibited) state, the N-terminal SH2 domain folds into the PTP domain, preventing substrate accessibility. Conformational changes follow after tyrosine phosphorylation of the kinase domain activation segment, leading to subsequent phosphatase activation [131]. In 2016, Novartis developed an allosteric, non-covalent SHP2 inhibitor SHP099. SHP099 stabilises SHP2 in its auto-inhibited conformation and suppresses SHP2-mediated ERK1/2 signalling (Figure 2B). This leads to the inhibition of proliferation of RTK-driven human cancer cells in vitro and reduction of tumour burden in mouse tumour xenograft models [132]. The effectiveness of SHP099 as a single agent in clinically relevant mouse models of AML reduced leukaemogenesis and leukaemic blast stemness [133].

This highlights how allosteric inhibition of PTPs holds promise in targeting oncogenic RTK signalling. However, several challenges remain when developing these inhibitors. Defining unique allosteric pockets is difficult due to the similarity in overall protein structure of PTPs. Because of their flexible structure, allosteric sites can shift or disappear depending on protein conformation. The weak binding activity of allosteric inhibitors can result in reduced potency, which requires a lot of optimisation to improve drug-likeness.

Taken together, PTPs represent promising therapeutic targets for the inhibition of oncogenic FLT3 ITD signalling. This may be achieved either by enhancing the activity of antagonistic PTPs, such as PTPRJ, or by suppressing the function of stimulatory PTPs, including SHP2. As outlined in this review, multiple strategies are currently being explored to modulate PTP activity with the goal of regulating signalling. However, substantial research is still required to develop effective PTP-targeting compounds for the treatment of AML patients harbouring FLT3 ITD mutations.

### 6.4. Dimerisation of PTPs

There is substantial evidence that many receptor protein tyrosine phosphatases (RPTPs), including RPTPα, GLEPP1, RPTPR, Sap-1, PTPRC, and RPTPε, undergo dimerisation in living cells, which plays a key role in regulating their activity (reviewed in detail by Bohmer, Weibrecht et al. [134,135]. The transmembrane domain and/or adjacent hydrophobic regions are often involved in this dimerisation process, as observed in RPTPBR7, PTP-SL, GLEPP1, Sap-1, and PTPRJ (Dep-1) [115,136,137,138,139,140]. Sequence alignment of the transmembrane domains of the 20 human RPTPs reveals a frequent presence of hydrophobic amino acids such as isoleucine and valine within the GxxxG motif—known as the Sternberg–Gullick motif—which promotes dimerisation [141,142]. In PTPRJ, a glycine zipper motif (GxxxGxxxG) facilitates close interaction between transmembrane helices, promoting dimer formation. These motifs are generally known to mediate helix–helix interactions and stabilise membrane-bound oligomers.

In addition to transmembrane regions, the extracellular domains of RPTPs also contribute to dimerisation, as shown in Sap1 [138], PTPRJ [143], LAR, and RPTPl [144]. Dimerisation affects RPTP activity by inducing conformational changes that can block access to the active site. This was first demonstrated in RPTPα, where one monomer inserts a helix-loop-helix wedge into the catalytic site of its dimer partner, inhibiting substrate access [134,145]. Similar inhibitory mechanisms have been proposed for other RPTPs and cytosolic PTP1B homodimers [144].

Dimerisation can also be regulated by extracellular ligands, as in the case of RPTPζ [106], or by oxidative modifications, such as those seen in RPTPα, where changes in the D2 domain stabilise dimer formation [146,147]. Although all domains of RPTPs may contribute to dimerisation, the transmembrane domain plays a particularly critical role in regulating activity [139,148,149]. For example, mutations disrupting the glycine zipper motif in PTPRJ reduce its oligomerisation, which in turn enhances phosphatase activity and suppresses EGFR-driven cancer phenotypes [116,139]. These mutations also reduce the phosphorylation of FLT3 (both WT and ITD forms) and impair downstream signalling and cell proliferation, indicating increased phosphatase activity [116].

Peptide agonists (Figure 3B) and monoclonal antibodies targeting the PTPRJ ectodomain can disrupt dimerisation and enhance its activity, and ligand binding (e.g., TSP1) has also been shown to activate PTPRJ [27,143,150]. These findings suggest that promoting RPTP activity—particularly those counteracting oncogenic kinases such as FLT3 ITD—may represent a promising therapeutic strategy for diseases such as AML.

Importantly, the effects of dimerisation on RPTP activity vary across different RPTPs. For instance, the CD45RO isoform of PTPRC forms homodimers that suppress T-cell receptor signalling [151], while GLEPP1 dimers show reduced activity toward its putative substrate, TrkC [137]. These opposing outcomes underscore the need to investigate the functional consequences of dimerisation individually for each RPTP.

### 6.5. Redox Regulation of PTPs

A critical post-translational mechanism that regulates PTP activity is the oxidation of the catalytically essential nucleophilic cysteine residue. This cysteine is essential for the nucleophilic attack on the phosphate group of phosphor tyrosines. It exists in a thiolate (−S^−^) state, which is highly susceptible to oxidation (Figure 3C). Exposure to ROS such as hydrogen peroxide (H_2_O_2_) results in the conversion of this thiolate to sulfenic acid (−SOH). Low to moderate levels of ROS are integral to normal cell signalling and homeostasis. Cells generate transient bursts of ROS, which reversibly inhibit PTP, ensuring enhanced or sustained tyrosine phosphorylation [152]. Under mild oxidative conditions, this oxidation is reversible, allowing enzymatic activity to be restored by cellular reducing systems such as thioredoxin (Trx) and thioredoxin reductase (TrxR). Studies have shown that TrxR1 can directly interact with oxidation intermediates of PTP1B, facilitating its reactivation during H_2_O_2_ exposure. This interplay underscores the importance of the cellular redox environment in modulating PTP activity and, consequently, signalling pathways [153]. Structural changes in the active site such as cyclic sulfenamide formation can happen following oxidation of the nucleophilic cysteine, and previously hidden residues can then be exposed, protecting the PTP from further oxidation and allowing oxidised cysteine and redox agent interaction [154]. This regulatory mechanism is particularly relevant under conditions of oxidative stress, with important implications for both normal physiology and disease. 

Under sustained or severe oxidative stress, sulfenic acid can be further oxidised to sulfinic (−SO_2_H) or sulfonic acid (−SO_3_H) forms, leading to irreversible PTP inactivation [155,156]. Oxidative inactivation of PTPs has been described as a contributory mechanism in the leukaemic transformation of FLT3 ITD-positive cells [157,158]. Oncogenic STAT5 signalling results in elevated NOX4 expression catalysing ROS production [29]. By oxidising PTPRJ, increased ROS impairs PTPRJ’s phosphatase activity and results in enhanced FLT3 ITD-mediated oncogenic signalling and cell transformation. Inactivation of NOX4 or the use of specific ROS quenchers restores PTPRJ activity and diminishes leukaemic transformation both in vitro and in vivo (Figure 3C) [28,29]. Quenching of cellular ROS by inhibition of reduced NADPH oxidases or by overexpression of catalase or peroxiredoxin-1 (Prx-1) similarly results in restoration of PTPRJ activity and impairs FLT3 ITD activities [158]. This highlights a pathogenic axis involving NOX4-ROS-PTPRJ in aggressive AML, suggesting PTPRJ and NOX4 as potential therapeutic targets.

It is worthwhile to mention that PTP oxidation was proposed as a promoter of dimerisation-induced inhibition of PTPs. Oxidation of Cys723 in RPTP-D2 leads to a conformational change in the PTP, which triggers rotation of the two monomers in the RPTPα dimer relative to each other, thus stabilising the dimer in an inactive conformation [159].

### 6.6. Bispecific Antibody–Aptamer Chimeras—Immunologic Approaches to Enforce RTK-PTP Interaction

Therapeutic bispecific antibodies targeting cell surface proteins are emerging as promising cancer therapies and are currently being used in clinical studies for the treatment of inflammatory diseases and different forms of cancers [151,160,161,162]. Li et al. presented a novel bispecific antibody–aptamer (Ab-Ap) chimera designed to inhibit RTK activity in cancer cells, shown in the example of the recruitment of PTPRJ to the RTK MET. The chimera employs a bispecific conjugate composed of a PTPRJ antibody and a DNA aptamer that specifically binds to the MET receptor, enabling dual-site engagement. The aptamer competes with hepatocyte growth factor (HGF) for binding to MET’s extracellular domain, thereby preventing receptor dimerisation and activation by trans-autophosphorylation. Simultaneously, the PTPRJ antibody recruits PTPRJ to MET facilitating its dephosphorylation, consequently diminishing MET downstream signalling. This Ab-Ap chimera displays synergistic targeting capabilities, improved stability and specificity, and reduced immunogenicity in comparison with bispecific antibody chimeras [30]. Thus, further development of this system has big potential in therapeutic approaches targeting RTK pro-oncogenic activity. Given the inhibitory effect of PTPRJ on FLT3, this strategy can be extended to target FLT3 oncogenic signalling by developing appropriate aptamers, or even by utilising other FLT3 counteracting PTPs, such as PTPRC (Figure 3D).

## 7. Targeting RTK Activity and AML by Modulating Genome-Wide Regulation of Gene Expression—Controlling Protein Acetylation and DNA Methylation

In addition to molecule-specific impacts, the control of global players affecting gene regulation is an attractive alternative to control FLT3 ITD-mediated cell transformation. In particular, epigenetic dysregulation, including altered histone acetylation and DNA methylation, plays a central role in AML development.

### 7.1. Zinc-Dependent Histone Deacetylases 

Acetylation, specifically histone acetylation, is a biological process that is regulated by histone acetyltransferases (HATs) and histone deacetylases (HDACs) and controls chromatin compaction/gene expression, protein activity and stability, and metabolism. Aberrant histone deacetylation, due to increased histone deacetylase (HDAC) activity and expression, often correlates with pathological gene repression and neoplastic transformation. HDACs are often overexpressed in AML cells resulting in the repression of tumour suppressor genes such as p21 or BIM, maintenance of leukaemia stem cells, and resistance to differentiation and apoptosis [163]. It has been demonstrated that FLT3 ITD enhances the activity of HDACs, which decrease acetylation at enhancer and promoter regions of oncogenic genes, such as *MYC*, *BCL2*, and *HOXA9* [164]. Consequently, this leads to open chromatin conformation and increased transcriptional activity of leukaemogenic genes. Thus, HDACs have been considered as important targets in cancer therapy [165]. In general, HDAC inhibitors (HDACis) result in histone hyperacetylation, chromatin relaxation, and reactivation of silenced genes. Consequently, they are reported to induce cell cycle arrest, apoptosis, reduced angiogenesis, and differentiation in cancer cells to combat these cells (recently reviewed by Zhang [34]. The induction of apoptosis in leukaemic blasts and promotion of differentiation (especially in M3/acute promyelocytic leukaemia) have been reported for HDACi treatment. Further, selective inhibition of HDAC1 induced degradation of FLT3 via inhibition of the chaperone function of HSP90 in AML cells [166]. HSP90 is acetylated and subsequently disassociates from FLT3, which is then polyubiquitinated and degraded by the proteasome [166,167].

In FLT3 ITD-positive AML, upregulated HDAC8 deacetylates and thereby inactivates p53, promoting leukaemia maintenance and resistance to TKIs [58,168,169]. Inhibition of HDAC8 reactivates p53. In xenograft models, the combination of FLT3 TKI (quizartinib) and HDAC8 inhibition (22d) reduces FLT3 ITD-positive cells and disease progression significantly [58]. The HDACi CUDC-907 targets FLT3 ITD signalling by acting at PI3K and reduces downstream signalling and leukaemogenesis. Thus, it results in the induction of apoptosis and overcoming of resistance. CUDC-907 treatment leads to increased acetylation of FLT3, which results in its degradation. These effects could be observed in vitro and in vivo. Phase 1 clinical trials showed that it is well tolerated, and toxicity is comparable or even better than that of FDA-approved single-target drugs [35]. A recent study revealed that the novel class I HDACi HCH9033 synergised with FLT3 inhibitor quizartinib and rescued quizartinib resistance in FLT3 ITD AML via enhancing DNA damage response [170]. Several studies show that the FLT3 degradation process and apoptosis of AML cell lines are enhanced by the combination of HDACis with FLT3 TKIs [171,172]. Conclusively, acetylation modulators in combination with conventional therapy have been shown to be very promising agents in improving treatment options in addition to HDACi or TKI alone. Despite several preclinical studies, mostly in combination therapies, currently, there are no HDAC inhibitors that are FDA-approved specifically for treating AML.

### 7.2. NAD-Dependent Histone Deacetylases 

It has been elucidated that sirtuins, a group of 7 (SIRT1-7) NAD^+^-dependent HDACs, also play critical and context-dependent roles in AML (recently reviewed by Strzałka [173]). Unlike classical HDACs, sirtuins belong to Class III HDACs and are involved in epigenetic regulation, metabolism, DNA repair, and stemness—all highly relevant to AML pathogenesis and progression. However, the molecular mechanisms of how the activity of sirtuins is transferred to cell physiologic responses is context dependent and needs further exploration [174].

It has been demonstrated that SIRT1 promotes leukaemic cell survival by deacetylating and inactivating tumour suppressors such as p53, thus inhibiting apoptosis [175]. Inhibiting SIRT1, either pharmacologically or via RNAi-mediated knockdown, restores p53 activity and with that the sensitivity of FLT3-mutated AML cells to TKI treatment and chemotherapy [176]. It was shown that mitochondria-localised SIRT3 inhibits chemotherapy-induced mitochondrial ROS production and increases oxidative phosphorylation in AML. Inhibition of SIRT3 worked synergistically with cytarabin and led to leukaemic blast destruction both in vitro and in mouse models [177]. In contrast, it can inhibit p53 degradation by negatively regulating MDM2 transcription via PTEN [178].

Furthermore, high H3K18 deacetylation by SIRT7 enhances persistence of the malignant phenotype of cancer cells [179,180]. In addition, SIRT7 attenuates the transcription of specific tumour-suppressor genes and induces rRNA production for the metabolic needs of the cancer cell [179,181]. In most cases, SIRT7 is well known for its tumour-promoting functions. However, in haematologic malignancies including AML, the protein seems to act differently. Raza U. et al. described the effect of SIRT7 on nuclear respiratory factor 1 (NRF1) in haematopoietic stem cells, which involves inhibition of transcription of ribosomal proteins. This reduces stress and enables the maintenance of a pool of HSCs, which in turn protect the cells from leukaemogenesis [182]. Thus, SIRT7 links H3K18 deacetylation to the maintenance of oncogenic transformation [179].

In general, SIRT7 expression levels in leukocytes in patients with AML and CML are reduced. A gene expression analysis in AML patients without cytogenetic abnormalities showed that low SIRT7 mRNA expression was associated with shorter overall survival, especially in the FLT3 ITD-mutated subgroup [183]. We demonstrated that low SIRT7 expression correlates with poor prognosis of AML patients [184]. Impaired expression of SIRT7 is observed in AML and CML compared to healthy donors. This suppression was disease related: remission with a positive response to treatment results in increased levels of SIRT7, while disease progression or relapse is associated with a subsequent decrease in the SIRT7 level. Thus, it could be speculated that oncogenic FLT3 ITD in AML samples or Bcr-Abl in CML expand their oncogenic activity by downregulation of this deacetylase. The C/EBPalpha transcription factor, which is suppressed in FLT3 ITD-positive cells, controls the expression of the SIRT7 gene. Consequently, the suppression of expression is dependent on the aberrant kinase activity of oncogenic FLT3 ITD [184]. In addition, SIRT7 expression is higher in patients who achieved complete remission before mobilisation compared to those who demonstrated partial remission [185]. Taken together, a potential tumour-suppressive role of SIRT7 in haematologic malignancies can be proposed. In this case, a specific activation of Sirt7 in the leukaemic blast cells might be a possible therapy avenue.

Although very promising, the cell type and cancer specific intricacies of (de)acetylation and the corresponding enzymes are still topics of ongoing research. Understanding them in the context of specific cell physiology and continuing to explore their potential in clinical trials and their efficacy in combination therapies remains crucial. Several HDACis have already been approved for use in clinical treatments. Notably, HDACis have been shown to be more effective in treating haematological malignancies than solid tumours, highlighting their promising role in the treatment of FLT3-mutant AML [186,187].

### 7.3. DNA Methyltransferases

Aside from aberrant acetylation, epigenetic dysregulation of DNA methylation is also a key factor in the progression of myelodysplastic syndromes (MDSs) to AML [59,188]. AML is often characterised by global DNA hypomethylation leading to genomic instability and hypermethylation at specific promoters, particularly of tumour suppressor genes (e.g., *CDKN2B*, *p15INK4B*). In addition, loss-of-function mutations of DNA methyltransferases (DNMTs) are common in 20–30% of AML cases (recently reviewed by Wang, 2025; [36]. These changes contribute to abnormal self-renewal, proliferation, and blocked differentiation of haematopoietic cells.

Ten-eleven translocation 2 (TET2) dioxygenase catalyses 5-hydroxymethylcytosine (5hmC) production and is involved in DNA demethylation. TET2 exhibits a relatively high mutation frequency (12–34%) in AML patients [189,190], and deletion of TET2 causes haematologic malignancies in mice [191]. Furthermore, isocitrate dehydrogenases 1 and 2 (IDH1/2) are metabolic enzymes that catalyse the reversible oxidative decarboxylation of isocitrate to yield α-ketoglutarate, which inhibits TET2 and consequently results in DNA hypermethylation. Somatic mutations in the IDH1 and IDH2 genes are found in about 15–20% of AML patients [192,193,194]. This prevalence highlights the impact of global DNA methylation in AML and an opportunity for new treatments, including FLT3 ITD-positive AML.

Currently, nucleoside DNMT inhibitors such as azacytidine and decitabine have been approved by the FDA and EMA for the treatment of AML and further myeloproliferative malignancies. In particular, elderly or unfit AML patients not eligible for intensive chemotherapy or AML with myelodysplasia-related changes are treated with DNMT inhibitors. However, these therapies are usually not curative, and due to the gradual onset of effect, resistances may develop [36]. Various combination therapies of the DNMT inhibitor azacitidine with BCL2 inhibitors, HDACi, kinase inhibitors, metabolic enzyme inhibitors, monoclonal antibodies, immune checkpoint inhibitors, and anti-apoptotic protein inhibitors are established (as reviewed by Li 2025, [59]. Azacitidine plus venetoclax is a standard of care for patients with newly diagnosed AML who are unfit for intensive chemotherapy. Here, venetoclax sensitises AML cells to apoptosis, and hypomethylating agents relieve methylation-driven resistance. Additionally, DNMT3A- [195,196] and TET2-targeted therapies [197,198] are under development.

The further understanding and targeting of aberrant cellular acetylation or methylation profiles for the treatment of FLT3 mutant AML are promising approaches to suppress leukaemic development. To clarify if these systems will be sufficient to diminish FLT3 ITD-driven oncogenic cell transformation and efficient enough to eradicate leukaemic cell clones completely, further studies are needed.

## 8. Conclusions

FLT3, as an RTK, directs haematologic cell proliferation and differentiation. Mutations in the FLT3 gene, namely, FLT3 ITD and FLT3 TKD, lead to constitutive and ligand-independent activation of the kinase driving leukaemic cell transformation in AML. Current treatments target oncogenic FLT3 activity but are often linked to resistance development. The persistent challenge of relapse and resistance in FLT3-mutated AML underscores the need for novel therapeutic approaches beyond current TKIs [41,199,200]. Exploring new treatment options is vital to improve treatment outcomes for AML patients with FLT3 mutations. A deeper mechanistic understanding of mutant FLT3 regulation, including the role of protein tyrosine phosphatases (PTPs), altered FLT3 biogenesis, and downstream signalling pathways, offers promising avenues for intervention. Targeting leukaemic stem cells and exploiting vulnerabilities in aberrant signalling networks as well as epigenetic systems may enhance treatment durability. Future research should prioritise the development of combination strategies that integrate FLT3 inhibition with agents targeting complementary pathways to overcome resistance mechanisms, address the problem of leukaemic clones, and thus improve clinical outcomes.

## Figures and Tables

**Table 1:** Investigated and potentially suitable approaches for directly targeting oncogenic FLT3 ITD activity.

Target	Mode of Action	Agent	References
Kinase activity	Inhibition	FLT3-specific TKIs (midostaurin, gilteritinib, quizartinib, crenolanib)	reviewed in [12]
		Multi-kinase-targeting TKIs (cabozantinib, dasatinib, mivavotinib)	[13]
		ROS quenchers (diphenyleneiodonium (DPI), N-acetylcysteine)	[14]
Glycosylation/surface expression	Inhibition	Statins (fluvastatin)	[15]
		2-DG	[16]
		Tunicamycin	[17]
	Promotion	TKIs (midostaurin, sorafenib, quizartinib)	[18,19]
		Valproic acid	[20]
Degradation	Promotion	Inhibitors of deubiquitination enzymes (thiolutin, HBX19818, P22077, WP1130, EOAI3402143)	[21,22]
		Stimulators of E2-conjugating enzymes/E3 ligases (decursin)	[23]
		Inhibitor of HSP90 (17-AAG)	[24]
		PROTACs (quizartinib-based, dovitinib-based, pomalidomide, A2, LWY713, FLT3 PROTAC molecule 35, A20, gilteritinib-based B3-2)	[25]
Dimerisation (of TRK, EGFR, PDGFR) *	Inhibition	Gangliosides (GM1, modification of endogenously expressed gangliosides GM2, GD1a, GD1b, GD3, GT1b)	[26]

* Targeting approach has not yet been explored in modulation of FLT3 activity, but it represents a promising strategy for therapeutic intervention.

**Table 2:** Investigated and potentially suitable approaches to targeted modulators of FLT3 ITD activity.

Target	Mode of Action	Agents	References
Tumour-suppressive PTPs *	Promoting activation	Natural ligands (TSP1), peptide agonists (PTPRJ-pep5, PTPRJ-pep19, PTPRJ-pep23, PTPRJ-pep24)	[27]
	Inhibition of deactivation	ROS quencher (schisandrin B)	[28,29]
	Promoting interaction	Bispecific antibody-aptamer chimeras (PTPRJ-MET)	[30]
Pro-oncogenic PTPs	Promoting degradation	PROTACs (SHP2-D26)	[31]
	Inhibition of activity	Allosteric inhibitors (SHP099)	[32]
		Orthosteric inhibitors (PHPS1)	[33]
Acetylation/methylation	Inhibition of histone deacetylases (HDACs) activity	HDAC inhibitors (multiple inhibitors, CUDC-907)	[34,35]
	Inhibition of methyltransferases	Nucleoside DNMT inhibitors (azacytidine and decitabine)	[36]

* Targeting approaches were explored for PTPRJ but may be applicable for other FLT3 activity-suppressive PTPs.

**Table 3:** Combinatorial approaches to treat FLT3 ITD-mutated AML.

No.	Target	Mode of Action	Agent	Reference
1.	FLT3 kinase activity	Inhibition	TKI	[46]
	BCL-2	Inhibition	Venetoclax	
2.	FLT3 kinase activity	Inhibition	TKI	[17]
	FLT3 glycosylation	Inhibition	Fluvastatin, tunicamycin	
3.	FLT3 kinase activity	Inhibition	TKI	[58]
	HDAC activity	Inhibition	HDAC8 inhibitor 22d, HDACi HCH9033, 17-AAG, panobinostat	
4.	FLT3 kinase activity	Inhibition	TKI	[59]
	Methyltransferase activity	Inhibition	Azacitidine	
5.	FLT3 degradation	Promotion	Decursin	[23]
	BCL-2	Inhibition	Venetoclax	
6.	FLT3 degradation	Promotion	PROTAC	[60]
	BCL-2	Inhibition	Venetoclax	
